# Supplementary material for: CircDOCK1 Regulates miR-186/DNMT3A to Promote Osteosarcoma Progression
Source: Biomedicines. 2022 Nov 23;10(12):3013. doi: 10.3390/biomedicines10123013 (PMC9775081; doi:10.3390/biomedicines10123013)
Supplement: Supplementary file 1 [file biomedicines-10-03013-s001.zip › biomedicines-2009884-supplementary.pdf]

Supplementary Table S1. Primer sequences in this study

CircDOCK1	Forward	5’- CCAGAGGCACGTCCAGATTA -3’
	Reverse	5’- AGGAAACTCCGCGTCTAGG -3’
miR-186	Forward	5’- TTAATTCCGATAACGAACGAGA -3’
	Reverse	5’- CGCTGAGCCAGTCAGTGTAG -3’
DNMT3A	Forward	5’- TATGAACAGGCCGTTGGCATC -3’
	Reverse	5’- AAGAGGTGGCGGATGACTGG -3’
GAPDH	Forward	5’- CCTGCCGGTGACTAACCCTG -3’
	Reverse	5’- TCCACCACTGACACGTTGGC -3’
